# A retrospective study comparing oral celecoxib combined with flurbiprofen hydrogel patch versus oral celecoxib therapy for lateral epicondylitis: real-world analysis of the anti-inflammatory and analgesic efficacy and safety

**DOI:** 10.3389/fphar.2026.1774834

**Published:** 2026-04-10

**Authors:** Jinying Wang, Shiqiang Zhuo, Yongfeng Li, Junjie Zheng, Qinci Xie, Lin Huang, Xiaofeng Chen, Pei-Chun Hsu, Gaofeng Huang

**Affiliations:** 1 Department of Orthopedics, Jinjiang Municipal Hospital (Shanghai Sixth People’s Hospital Fujian), Jinjiang, Fujian, China; 2 Clinical Research Center for Orthopedic Trauma and Reconstruction of Fujian Province, Jinjiang, Fujian, China; 3 Department of Orthopedics, Shanghai Sixth People’s Hospital affiliated to Shanghai Jiao Tong University School of Medicine, Shanghai, China; 4 Key Laboratory of Neuroregeneration of Jiangsu and Ministry of Education, Coinnovation Center of Neuroregeneration, Medical School, Nantong University, Nantong, China

**Keywords:** anti-inflammatory, Bone and joint disease, celecoxib, flurbiprofen hydrogel patch, lateral epicondylitis

## Abstract

**Objective:**

The study is aimed to retrospectively compare the efficacy, safety, and impact on serum inflammatory markers between oral celecoxib combined with flurbiprofen hydrogel patch and oral celecoxib alone in the treatment of lateral epicondylitis.

**Methods:**

A retrospective cohort analysis was conducted on 64 patients diagnosed with lateral epicondylitis between January 2022 and December 2024. Patients were divided into two groups: the combined therapy group (oral celecoxib + topical flurbiprofen hydrogel patch, n = 32) and the single therapy group (oral celecoxib alone, n = 32). The primary outcome measures were Visual Analog Scale (VAS) scores and Patient-Rated Tennis Elbow Evaluation (PRTEE) scores measured at pre-treatment, 2 weeks, and 4 weeks of treatment. Secondary outcome measures included overall efficacy rate, effecting time, and 3-month recurrence rate. Serum inflammatory markers (tumor necrosis factor-alpha (TNF-α), interleukin-6 (IL-6), and C-reactive protein (CRP)) were measured and compared between the two groups at pre-treatment, 2 weeks, and 4 weeks. Adverse events were evaluated between two groups.

**Results:**

Pre-treatment characteristics were comparable between the two groups. Regarding clinical efficacy, at both 2 and 4 weeks post-treatment, the combined therapy group showed significantly lower VAS scores (2 weeks post-treatment: 3.9 ± 1.4 vs. 5.0 ± 1.5, P = 0.003; 4 weeks post-treatment: 2.2 ± 1.1 vs. 3.6 ± 1.3, P < 0.001) and PRTEE scores (2 weeks post-treatment: 37.8 ± 10.5 vs. 46.3 ± 12.1, P = 0.004; 4 weeks post-treatment: 21.0 ± 9.8 vs. 31.4 ± 10.9, P < 0.001) compared to the single therapy group. The combined therapy group also demonstrated a higher overall efficacy rate (81.3% vs. 59.4%, P = 0.049) and a faster effecting time (median: 4.0 days vs. 7.0 days, P = 0.003). Regarding inflammatory markers, at both 2 and 4 weeks post-treatment, serum levels of TNF-α, IL-6, and CRP in the combined therapy group were significantly reduced from pre-treatment. Specifically, at 4 weeks post-treatment, TNF-α levels were significantly lower in the combined therapy group (12.3 ± 3.5 pg/mL vs. 16.8 ± 4.1 pg/mL, P < 0.001), as were IL-6 levels (8.1 ± 2.9 pg/mL vs. 11.7 ± 3.3 pg/mL, P < 0.001) and CRP levels (5.2 ± 2.1 mg/L vs. 8.0 ± 2.8 mg/L, P < 0.001). In terms of safety, there was no significant difference in the overall incidence of adverse events (21.9% vs. 12.5%, P = 0.327) and the 3-month recurrence rate (15.6% vs. 34.4%, P = 0.085) between two groups.

**Conclusion:**

For lateral epicondylitis, the combination of oral celecoxib and topical flurbiprofen hydrogel patch is superior to oral celecoxib alone in alleviating pain, improving function, and reducing systemic inflammation. The combined therapy also increases the overall efficacy rate, reduces the effecting time, without increasing systemic adverse events. The combined regimen represents an effective and safe treatment option to offer benefits for clinical application.

## 1 Introduction

Lateral epicondylitis, also known as tennis elbow, is a chronic overuse condition characterized by pain and tenderness at the lateral epicondyle of the humerus (Lenoir et al., 2019). Its underlying pathology is a degenerative tendinopathy of the common extensor origin, particularly the extensor carpi radialis brevis tendon, accompanied by local non-infectious inflammatory response (Ahmad et al., 2013). With a prevalence of approximately 1%–3% in the general population, it predominantly affects individuals engaged in repetitive wrist extension activities, significantly impairing daily life and occupational function (Johns and Shridhar, 2020).

Current management follows a stepwise approach, with core goals of pain relief, inflammation control, tissue repair promotion, and functional recovery. Non-steroidal anti-inflammatory drugs (NSAIDs), owing to their effective anti-inflammatory and analgesic properties, are widely recommended as first-line conservative treatment (Ahmed et al., 2023). Among them, oral selective COX-2 inhibitors can effectively control systemic inflammation and alleviate pain while posing a relatively lower risk of gastrointestinal adverse events. However, oral administration alone may result in systemic drug distribution with potentially insufficient drug concentration at the lesion site (Duncan et al., 2019).

Over the past years, topical NSAID formulations have gained increasing attention in the management of chronic musculoskeletal pain (Green et al., 2002). Flurbiprofen hydrogel patch, a novel topical non-steroidal anti-inflammatory agent, exerts significant anti-inflammatory and analgesic effects primarily by inhibiting the production and release of prostaglandins, demonstrating confirmed efficacy in various acute and chronic inflammatory conditions and pain management, such as periarthritis of the shoulder and knee osteoarthritis (Sánchez-Milá et al., 2025). Their advantage lies in delivering the drug directly to the affected area, achieving high local concentrations while markedly reducing the risk of systemic adverse events associated with substantial systemic absorption. Theoretically, combining oral celecoxib with topical flurbiprofen hydrogel patch may offer a superior pharmacological strategy for lateral epicondylitis through a synergistic mechanism of systemic anti-inflammatory analgesia and local targeted reinforcement (Pattanittum et al., 2013).

Despite exploratory use of this combination in clinical practice, high-quality real-world evidence supporting its clear advantage over single therapy with oral agents remains scarce. Previous studies have largely focused on single-drug therapies or physical interventions, leaving a paucity of controlled research, especially analyses based on actual clinical practice data, regarding the efficacy and safety of combined oral and topical NSAIDs. Therefore, to address this evidence gap and guide rational clinical pharmacotherapy, this study aims to conduct a retrospective cohort analysis within a real-world clinical setting. We will compare the clinical efficacy and safety of oral celecoxib combined with flurbiprofen hydrogel patch versus oral celecoxib alone for the treatment of chronic lateral epicondylitis. The study will focus on evaluating differences between the two regimens in improving pain intensity, inflammation control, elbow function, overall efficacy rate, effecting time, recurrence rate and adverse events. Emerging evidence suggests that low-grade systemic inflammation plays a crucial role in the pathogenesis and progression of lateral epicondylitis. In particular, pro-inflammatory cytokines such as tumor necrosis factor-alpha (TNF-α) and interleukin-6 (IL-6), along with the acute-phase reactant C-reactive protein (CRP), have been implicated in the chronic inflammatory processes underlying tendinopathy (Wu J et al., 2025). Recent studies have further highlighted the clinical relevance of these biomarkers, demonstrating that elevated serum levels of TNF-α and IL-6 are associated with greater pain intensity, functional impairment, and poorer treatment outcomes in patients with LE (Farì et al., 2025; Lei et al., 2025). Moreover, monitoring changes in these inflammatory markers has been proposed as a valuable tool for assessing therapeutic response and guiding personalized treatment strategies (Cortella et al., 2025). However, despite the growing recognition of their pathophysiological role, few studies have systematically compared how different conservative treatment modalities modulate these systemic inflammatory markers alongside clinical outcomes. The findings are expected to provide robust data support and clinical reference for optimizing the pharmacological management of this condition.

## 2 Methods

### 2.1 Study design and participants

This was a retrospective cohort study in a real-world clinical setting to evaluate the efficacy and safety of two treatment groups. The study population consisted of patients diagnosed with lateral epicondylitis who visited the Orthopedics outpatient clinics between 1 January 2022, and 31 December 2024. All data, including demographic information, clinical assessments, and serum laboratory results, were obtained from existing electronic medical records. No additional blood samples were collected for research purposes. Following institutional policy, the study was exempt from ethics committee approval and the requirement for informed consent was waived.

### 2.2 Patient inclusion and exclusion criteria

Inclusion criteria: ① Age 18–65 years; ② Patients met the clinical diagnostic criteria for lateral epicondylitis (localized tenderness over the lateral epicondyle and positive Mill’s test) (Wolf, 2023); ③ Disease duration ≥4 weeks; ④ Medical records indicated continuous treatment with either the combined therapy or single therapy protocol for at least 4 weeks; ⑤ Complete demographic data, clinical assessment records, and serum inflammatory marker, including TNF-α, IL-6, CRP test results from pre-treatment, 2 weeks, and 4 weeks of treatment were available.

Exclusion criteria: ① History of elbow fracture or surgery, or presence of other joint diseases such as rheumatoid arthritis or gout; ② Severe cardiac, hepatic, or renal insufficiency; allergy to celecoxib or flurbiprofen; or presence of systemic inflammatory joint diseases, such as rheumatoid arthritis, gout, spondyloarthritis, or other autoimmune arthropathies; ③ Receipt of other systemic treatments during the study period, such as local corticosteroid injections, shockwave therapy, or acupuncture; ④ Pregnancy or lactation; ⑤ Missing key clinical or laboratory data.

### 2.3 Treatment and grouping

In our real-world setting, treatment allocation was determined by the attending physicians based on comprehensive clinical judgment, including disease severity, prior treatment response, patient preference, and economic considerations. Based on the actual treatment received, patients were divided into two groups: (1) Combined therapy group: oral celecoxib capsules (specification: 200 mg) once daily, combined with topical application of flurbiprofen hydrogel patch (specification: 40 mg/patch) once daily, applied to the point of maximal tenderness on the lateral aspect of the elbow. Treatment continued for four consecutive weeks. (2) Single therapy group: oral celecoxib capsules only, specification and dosage same as the combination group, without the use of any other topical NSAIDs or analgesic patches. Treatment continued for four consecutive weeks.

### 2.4 Outcome measures

Demographic information from the two groups were recorded, including age, gender, dominant hand of the affected side, disease duration and body mass index (BMI).

Primary outcome measures: (1) Pain: assessed using the Visual Analog Scale (VAS, 0-10) recorded at pre-treatment, 2 weeks, and 4 weeks of treatment. (2) Function of the elbow: assessed using the Patient-Rated Tennis Elbow Evaluation questionnaire (PRTEE scores, 0-100) recorded at the same time points. The PRTEE questionnaire used in our study is the officially validated Chinese version, which has undergone cross-cultural adaptation and psychometric testing.

Secondary outcome measures: Overall efficacy rate (defined as a ≥50% reduction in VAS score from pre-treatment at 4 weeks), effecting time (time to ≥20% reduction in VAS score), and 3-month recurrence rate (revisitation for the same pain after symptom resolution). Laboratory tests: venous blood samples were collected from patients at pre-treatment, 2 weeks, and 4 weeks of treatment. Serum levels of TNF-α, IL-6, and CRP were analyzed. Serum CRP levels were measured using an immunoturbidimetric assay on an automated clinical chemistry analyzer, following the manufacturer’s protocols. The detection limit was 0.1 mg/L, with intra- and inter-assay coefficients of variation (CVs) of <3% and <5%, respectively. Serum TNF-α and IL-6 concentrations were quantified using commercially available enzyme-linked immunosorbent assay kits, according to the manufacturer’s instructions. All samples were assayed in duplicate to ensure accuracy. The minimum detectable concentrations were 0.5 pg/mL for TNF-α and 0.2 pg/mL for IL-6. The intra-assay CVs were <5% for both cytokines, and inter-assay CVs were <8% (Wang S et al., 2025). Adverse events occurring during the treatment period were recorded, including gastrointestinal reactions, local skin reactions, and headache or dizziness.

### 2.5 Statistical analysis

Data analysis was performed using SPSS software. Normally distributed continuous data are presented as mean ± standard deviation (Mean ± SD), and between-group comparisons were made using independent samples t-tests. Non-normally distributed data are presented as median and interquartile range [M (Q1, Q3)], and between-group comparisons were made using the Mann-Whitney U test. Categorical data are presented as number (percentage), and between-group comparisons were made using the chi-square test or Fisher’s exact test. P < 0.05 was considered statistically significant.

## 3 Results

From the initial 85 patients of lateral epicondylitis receiving these treatment, 21 patients were excluded, including (12 patients receiving steroid injection, seven patients with incomplete medical history, and two patients with other elbow joint diseases). Finally, 64 patients were included in this study, with 32 each in the two groups. No statistically significant differences were observed between the two groups regarding demographic characteristics, disease duration, or pre-treatment levels of pain and function of the affected elbow (P > 0.05). These findings confirm that the groups were well-matched and comparable before treatment (Table 1).

**TABLE 1:** Demographic information of the patients from combined therapy and single therapy groups.

Demographic parameter	Combined therapy group (n = 32)	Single therapy group (n = 32)	Statistics	P value
Age, M±SD	46.5 ± 9.8	47.2 ± 8.4	t = 0.307	0.76
Gender, n (%)	​	​	χ^2^ = 0.127	0.722
Male	15 (46.9%)	14 (43.8%)	​	​
Female	17 (53.1%)	18 (56.2%)	​	​
Dominant hand/affected side, n (%)	25 (78.1%)	23 (71.9%)	χ^2^ = 0.337	0.562
Disease duration, M (Q1, Q3)	5.0 (3.0, 8.0)	6.0 (3.8, 8.3)	Z = 0.829	0.407
BMI (kg/m^2^), M±SD	24.5 ± 2.9	23.9 ± 3.1	t = 0.807	0.423

A significant reduction in VAS scores was observed over time in both groups. However, the magnitude of pain reduction was significantly greater in the combined therapy group compared to the single therapy group (Table 2). The combined therapy group demonstrated significantly superior improvement in elbow joint function compared to the single therapy group at both 2-week and 4-week treatment time points (Table 3).

**TABLE 2:** VAS score of the patients from combined therapy and single therapy groups.

Time	Combined therapy group (n = 32)	Single therapy group (n = 32)	Differences (95% CI)	P value
Before treatment	7.4 ± 1.2	7.1 ± 1.3	0.3 (−0.3, 0.9)	0.332
Two weeks post-treatment	3.9 ± 1.4	5.0 ± 1.5	−1.1 (−1.8, −0.4)	0.003
Four weeks post-treatment	2.2 ± 1.1	3.6 ± 1.3	−1.4 (−2.0, −0.8)	<0.001

**TABLE 3:** PRTEE score of the patients from combined therapy and single therapy groups.

Time	Combined therapy group (n = 32)	Single therapy group (n = 32)	Differences (95% CI)	P value
Before treatment	70.2 ± 11.5	67.8 ± 12.8	2.4 (−3.6, 8.4)	0.427
Two weeks post-treatment	37.8 ± 10.5	46.3 ± 12.1	−8.5 (−14.1, −2.9)	0.004
Four weeks post-treatment	21.0 ± 9.8	31.4 ± 10.9	−10.4 (−15.7, −5.1)	<0.001

Before treatment, serum levels of TNF-α, IL-6, and CRP showed no statistically significant differences between the two groups (P > 0.05). Both treatment protocols significantly reduced the levels of these inflammatory markers. Notably, at post-treatment 2 and 4 weeks, serum levels of TNF-α, IL-6, and CRP were significantly lower in the combined therapy group than in the single therapy group (P < 0.05) (Tables 4–6). These results suggest that the combined therapy led to a more rapid and pronounced reduction in inflammatory markers.

**TABLE 4:** Serum TNF-α levels (pg/mL) of the patients from combined therapy and single therapy groups.

Time	Combined therapy group (n = 32)	Single therapy group (n = 32)	P value	P value (vs. Baseline)
Before treatment	25.4 ± 6.1	24.8 ± 5.9	0.682	-
Two weeks post-treatment	16.7 ± 4.3	20.1 ± 4.9	0.003	<0.001
Four weeks post-treatment	12.3 ± 3.5	16.8 ± 4.1	<0.001	<0.001

**TABLE 5:** Serum IL-6 levels (pg/mL) of the patients from combined therapy and single therapy groups.

Time	Combined therapy group (n = 32)	Single therapy group (n = 32)	P value	P value (vs. Baseline)
Before treatment	18.2 ± 5.2	17.6 ± 4.8	0.632	-
Two weeks post-treatment	10.5 ± 3.4	13.9 ± 3.8	<0.001	<0.001
Four weeks post-treatment	8.1 ± 2.9	11.7 ± 3.3	<0.001	<0.001

**TABLE 6:** Serum CRP levels (mg/L) of the patients from combined therapy and single therapy groups.

Time	Combined therapy group (n = 32)	Single therapy group (n = 32)	P value	P value (vs. Baseline)
Before treatment	15.8 ± 4.5	14.9 ± 4.1	0.398	-
Two weeks post-treatment	8.3 ± 2.7	10.6 ± 3.1	0.002	<0.001
Four weeks post-treatment	5.2 ± 2.1	8.0 ± 2.8	<0.001	<0.001

As for the overall efficacy rate, in the combined therapy group, 26 patients (81.3%) were considered clinically effective for the treatment, compared to 19 patients (59.4%) in the single therapy group. The overall efficacy rate was significantly higher in the combined therapy group (P < 0.05). For the effecting time, the median time was 4.0 days (IQR: 3.0–5.0 days) in the combined therapy group, which was significantly shorter than the 7.0 days (IQR: 5.0–9.0 days) observed in the single therapy group (P = 0.003).

At the 3-month follow-up, recurrence was reported in five patients (15.6%) from the combined therapy group and 11 patients (34.4%) from the single therapy group. Although the combined therapy group showed a trend toward a lower recurrence rate, this difference was non-statistically significant (P > 0.05).

For the adverse effect evaluation, no significant difference was found in the overall incidence of adverse events between the two groups. However, 4 cases of mild local skin reactions (itching or erythema) were observed in the combined therapy group, whereas none were reported in the single therapy group. All reported gastrointestinal adverse events were mild in severity, transient epigastric discomfort, mild acid reflux. No severe events occurred. Management included dose scheduling adjustments, such as taking medication after meals and/or short-term use of gastric mucosal protectants (Table 7).

**TABLE 7:** Adverse effects of the patients from combined therapy and single therapy groups.

Specific side effects	Combined therapy group (n = 32)	Single therapy group (n = 32)	P value (Fisher)
Gastrointestinal discomfort	2 (6.3%)	3 (9.4%)	0.5
Skin allergy	4 (12.5%)	0 (0.0%)	0.054
Headache/Dizziness	1 (3.1%)	1 (3.1%)	1
Total	7 (21.9%)	4 (12.5%)	0.327

## 4 Discussion

In this study, we found that compared with oral celecoxib alone, the combined therapy of oral celecoxib and topical flurbiprofen hydrogel patch for chronic lateral epicondylitis demonstrated significant advantages across multiple levels (Chen et al., 2025; Hou et al., 2025; Zhang et al., 2024; Fu et al., 2022). This combined regimen not only alleviated pain and improved elbow joint function more rapidly and significantly, but also more effectively reduced serum levels of systemic inflammatory markers, including TNF-α, IL-6, and CRP, without significantly increasing the overall risk of adverse reactions (Lu et al., 2025; Krishnan et al., 2024; Ji et al., 2024; Liu et al., 2020; Pitzer et al., 2014).

This study provides robust evidence for the systemic and local treatment strategy from both clinical and laboratory findings. Previous research indicated that lateral epicondylitis was not merely a localized tendinopathy. Its pain and dysfunction were often accompanied by a systemic low-grade inflammatory response (San et al., 2025; Pan et al., 2022; Song et al., 2021; Kahlenberg et al., 2015; Cohen and da Rocha Motta Filho, 2015). The therapeutic effects observed in this study can be better understood in light of recent advances in understanding celecoxib’s pharmacological mechanisms beyond conventional COX-2 inhibition. Emerging evidence has substantially expanded our knowledge of celecoxib’s anti-inflammatory and potential chondroprotective properties. A recent *in vitro* study demonstrated that celecoxib, particularly in combination with other agents, exerted anti-inflammatory effects through modulation of the nuclear factor (NF)-κB pathway, significantly reducing the expression and release of pro-inflammatory mediators including IL-6 and TNF-α in human osteoarthritic chondrocytes (Cheleschi et al., 2021). This mechanistic insight is particularly relevant to our findings, as the superior reduction in serum inflammatory markers in the combination therapy group may reflect enhanced suppression of NF-κB-mediated inflammatory cascades.

Furthermore, it revealed that celecoxib activated the nuclear factor erythroid 2-related factor 2 (Nrf2)/heme oxygenase-1 (HO-1) pathway, representing a novel antioxidant mechanism that contributed to its anti-inflammatory effects and promoted the recovery of damaged cartilage. The study demonstrated that celecoxib treatment significantly increased cell viability and reduced oxidative stress in chondrocyte injury models, with combination therapy showing superior efficacy compared to monotherapy (Shimasaki et al., 2024). This Nrf2/HO-1 pathway activation may partly explain the more pronounced reduction in systemic inflammatory markers observed in our combination therapy group, as oxidative stress and inflammation are intimately linked in tendinopathy pathogenesis.

The combination of oral celecoxib and topical flurbiprofen employed in our study aligns with the emerging concept of multi-target therapeutic approaches. The investigation in combination therapies in musculoskeletal conditions suggests that concurrent targeting of different inflammatory pathways may yield synergistic effects without compromising safety (Roberts et al., 2024). In this study, the superior improvement in VAS and PRTEE scores in the combined therapy group was associated with a more pronounced reduction in serum inflammatory markers. This suggests that the superiority of the combination protocol may stem from the dual anti-inflammatory mechanisms of oral celecoxib and topical flurbiprofen. The oral administration effectively controls the systemic inflammation, while transdermal delivery of flurbiprofen achieves high drug concentration at the lesion site, directly inhibiting prostaglandin synthesis and the release of inflammatory cytokines in local tissues (Wang Y et al., 2025; Al-Dhafer et al., 2021; Wang et al., 2020; da Luz et al., 2019). This synergistic effect may accelerate the resolution of local inflammation and improve the microenvironment for tissue repair, thereby manifesting as a faster onset of action (median time shortened by 3 days) and a higher overall efficacy rate at 4 weeks.

Regarding safety, the findings of this study align with the known pharmacological profiles of the drugs. There was no significant difference in the incidence of systemic adverse reactions, such as gastrointestinal effects between two groups. This reinforces the relative safety profile of celecoxib as a selective COX-2 inhibitor and the low systemic exposure characteristics of flurbiprofen hydrogel patch (Wu W et al., 2025; Scaturro et al., 2025; de Melo Viveiros et al., 2022; Xu et al., 2021). The observed local skin reactions in the combination therapy group (12.5%) represent a generally mild adverse effect, typically resolving without specific intervention and attributable to the direct topical application of the medication. Nonetheless, in clinical practice, patient education regarding potential local reactions and their management remains necessary.

Furthermore, concerning mid to long-term efficacy, although the reduction in the 3-month recurrence rate in the combined therapy group (15.6% vs. 34.4%) was not statistically significant within the limited sample size, it still suggests potential clinical relevance. More rapid control of acute inflammation and more complete resolution of initial symptoms may help break the cycle of pain-dysfunction, thereby potentially reducing the long-term risk of recurrence (Zhang et al., 2022; Zhu et al., 2021; Bazancir and Fırat, 2019; Dingemanse et al., 2014). Future studies with larger sample sizes and longer follow-up periods are required to validate this hypothesis.

This study has several limitations. Firstly, the retrospective design cannot avoid selection bias and information bias. Although strict inclusion/exclusion criteria were employed to minimize confounding factors, the allocation of treatment regimens was not randomized and may have been influenced by physician preference, patient compliance, and economic considerations. Our original exclusion criteria only excluded patients who received corticosteroid injections during the study period, without specifying a washout period. We recommend that future studies should establish a minimum washout period (≥3 months) to minimize confounding by prior treatments. The occupational and sports activity data would provide valuable context for interpreting outcomes. Unfortunately, due to the retrospective design of this study, detailed and systematic documentation of patients' occupational characteristics, sports participation, or activity modification during treatment was not available from the medical records. We understand this as an important missing variable that should be collected in future prospective studies. Secondly, the relatively small sample size may have affected the statistical power for secondary outcome measures, potentially preventing some clinically meaningful differences from statistical significance. This may have reduced statistical power for detecting significant differences in secondary outcomes (e.g., recurrence rate, adverse events) and may limit the generalizability of our findings. We also recommend future large-scale, multicenter prospective studies to validate our results. Thirdly, the observation period was relatively short, precluding assessment of longer-term maintenance of efficacy and functional joint outcomes. Finally, as this was a retrospective real-world study, we did not prospectively exclude patients based on gastrointestinal risk factors. However, we reviewed the baseline medical records and confirmed that no patient in either group had a documented history of active peptic ulcer disease or severe gastrointestinal conditions.

## 5 Conclusion

In conclusion, this real-world study demonstrates that the combination of oral celecoxib and topical flurbiprofen is a superior treatment option for lateral epicondylitis. Through synergistic anti-inflammatory effects, it outperforms oral single therapy in alleviating symptoms, improving function, and reducing inflammatory markers, with relatively good safety. It is recommended to carry out future prospective, large-sample randomized controlled trials to further confirm its long-term benefits and cost-effectiveness, thereby providing some higher-level evidences for clinical application and treatment.

## Data Availability

The original contributions presented in the study are included in the article/supplementary material, further inquiries can be directed to the corresponding authors.
